# Hospital contacts for injuries and musculoskeletal diseases among seamen and fishermen: A population-based cohort study

**DOI:** 10.1186/1471-2474-9-8

**Published:** 2008-01-23

**Authors:** Linda Kaerlev, Anker Jensen, Per Sabro Nielsen, Jørn Olsen, Harald Hannerz, Finn Tüchsen

**Affiliations:** 1 Research Unit of Maritime Medicine at University of Southern Denmark, Esbjerg, Denmark; 2 Linda Kærlev, Specialist in Occupational Medicine, Esbjerg, Denmark; 3 Danish Defence, Occupational Health Center South, Fredericia, Denmark/Department of Occupational Health, Haderslev Hospital, Denmark; 4 Department of Occupational Health, Esbjerg Hospital, Denmark; 5 School of Public Health, Department of Epidemiology, The Southern California Injury Center, UCLA, LA, USA; 6 National Institute of Occupational Health, Copenhagen, Denmark

## Abstract

**Background:**

We studied musculoskeletal diseases (MSD) and injuries among fishermen and seamen with focus on low back disorders, carpal tunnel syndrome (CTS), rotator cuff syndrome and arthrosis.

**Methods:**

Cohorts of all male Danish seamen (officers and non-officers) and fishermen employed 1994 and 1999 with at least six months employment history were linked to the Occupational Hospitalisation Register. We calculated standardised incidence ratios (SIR) for the two time periods, using rates for the entire Danish workforce as a reference.

**Results:**

Among fishermen, we found high SIRs for knee arthrosis, thoraco-lumbar disc disorders, injuries and statistically significant SIRs above 200 were seen for both rotator cuff syndrome and CTS. The SIR was augmented for injuries and reduced for hip arthrosis between the two time periods. The SIRs for injuries and CTS were high for non-officers. A sub-analysis revealed that the highest risk for CTS was found among male non-officers working as deck crew, SIR 233 (95% CI: 166–317) based on 40 cases. Among officers, the SIRs for injuries and MSDs were low. The number of employed Danish fishermen declined with 25% 1994–1999 to 3470. Short-term employments were common. None of the SIRs increased with increasing length of employment.

**Conclusion:**

Both fishermen and non-officers have high SIRs for injuries and fishermen also for MSD. Only the SIR for injuries among fishermen was augmented between 1994 and 1999. Our findings suggest an association between the incidence of rotator cuff syndrome and CTS and work within fishery. Long-term cumulative effects of employment were not shown for any of the disease outcomes. Other conditions may play a role.

## Background

Studies from many countries have claimed that the occurrence of lesions and musculoskeletal disorders (MSD) among fishermen is generally high, although substantial national differences in the working conditions of the fishing industry do exist [1-13]. In a recent study among fishermen in the United States, musculoskeletal symptoms causing work disruption in the past 12 months were reported by 38.5%, with low back symptoms accounting for 17.7%, followed by pain in the hands or wrists and shoulders, each location accounting for 7% of the cases [14]. In a recent pilot interview study in 2004 among 39 Danish fishermen, rotator cuff syndrome and carpal tunnel syndrome seemed to be common problems but mainly among the older fishermen (age above 50) [15]. Depending on job title on board, the fishermen reports handling the ice and the catch as major physical demanding factors especially in the past due to lack of automatisation. Although ice bands and band conveyors has been introduced on many vessels, some fishermen who work with ice in the hold still have physically demanding repetitive work with their shoulder elevated during work. Such working postures are known to be risk factors for damage to the shoulder tendons [16]. Fishermen doing repair on the net are known to have repetitive hyperflexing and twisting movements of the wrists, which are risk factors for development of carpal tunnel syndrome [17]. Finally fishermen have a high incidence of traumatic injuries to the shoulder or wrist during fishery.

Although recent studies have shown that heredity is a dominant factor in lumbar disc degeneration, heavy lifting and awkward working positions as well as obesity and joint injury may be modest risk factors for disc degeneration, and also for arthrosis of the knee and hip [18,19]. Factors augmenting the working conditions were bad weather conditions, small crews and long working hours [14,20,21]. A high frequency of work-related diseases and injuries has been reported in earlier studies of seamen [22-24].

Fishing methods have continuously evolved throughout recorded history and especially in the recent decades advances in mechanization of gear handling, improved performances of vessels and motorization, computer technology, navigation aids and fish detection have been seen. Similarly, the Danish merchant fleet has modern ships, staffed mainly by native officers and a growing number of foreign crew. We would expect that improvements in mechanization would be reflected in a decline in incidence rates for MSDs for seamen and fishermen. However, several studies of fishermen have identified methodological difficulty inherent in identifying the population at risk and in following them up [9].

We aimed to study whether hospitalizations for thoraco-lumbar disc disorders, arthrosis of the knee and hip, rotator cuff syndrome, carpal tunnel syndrome and injuries were more common among seafarers (by job type) and fishermen than for other economically active Danes, comparing two employment time periods, 1994 and 1999, and whether the standardised incidence ratios (SIR) increased with length of service.

## Methods

The present study population has already been described in detail [25,26]. Two five-year follow-up studies, one starting 1 January 1994 and the other 1 January 1999, were performed to examine potential changes over time in the SIRs of different diseases among Danish seamen and fishermen. A third follow-up study examined hospital contacts in the time period 1994–2003 as a function of length of service in the time period 1964–1993. Incidence rates were obtained by linking data from the Occupational Hospitalisation Register (OHR) to occupational cohorts extracted from the Danish Seafarer Registry (seamen) and from the fishing boat yearbooks, fishery yearbooks, the Danish Maritime Authority (DMA) files, tax and pension registries (fishermen) [23-28].

### Occupational cohorts

The cohort of Danish seamen was based upon individual data files kept at the Danish Seafarer Register. This data source is administered by the DMA and is regarded as almost complete and suitable for research purposes [27]. It is compulsory for the shipping companies to send a copy of the employment contract to the DMA each time a seaman signs on and off a Danish ship. Such information is also kept for retired or deceased seamen. The Registry has been computerised since 1986 and it includes information on name, birthday, job title, name and call signal of the ship, and dates of start and end of each employment period.

The fishermen cohort was established by using different data sources, which we believe cover all professional fishermen in the country. Company information was extracted from fishing boat yearbooks, fishery yearbooks, the DMA files, and tax files. The aim was to identify all fishing boats in the Danish fishing industry operating from 1989 to 1998, their owners and their company identification code. This code was used to extract employee data from the ATP Registry, the largest national pension scheme. The ATP holds data on the occupational history of each person employed on board all the registered fishing boats. It is compulsory for all companies in Denmark with employees working for 9 hours or more per week to participate in a non-contributory pension scheme. In addition, seamen and fishermen with a permanent address in Denmark also have a unique 10-digit Personal Identification Number (PIN), which has been assigned to each Danish resident since 1968. The number includes information on birthday and sex, and it is used by all authorities for registration purposes. We used the PIN to link the occupational data with each fisherman's or seaman's hospital contacts as an inpatient or outpatient as recorded by the nationwide Occupational Hospitalisation Register OHR [29].

### Inclusion criteria

A total of 36,113 officers and non-officers of either gender (persons with and without residence in Denmark) and 11,755 male fishermen with a PIN were initially retrieved from the files of the DMA and the pension registry. Due to a high turn-over of the cohorts, we restricted the cohorts to include only those employed at two baselines at 1 January 1994 and at 1 January 1999 together with information about their job history with length of service within the trades. Short-term employments were common: 30.6% of the seamen and 41.9% of the fishermen had in 1989–1998 a total length of service of less than 6 months. We therefore made restrictions for the length of service in the definition of the occupational groups.

**Figure 1:**
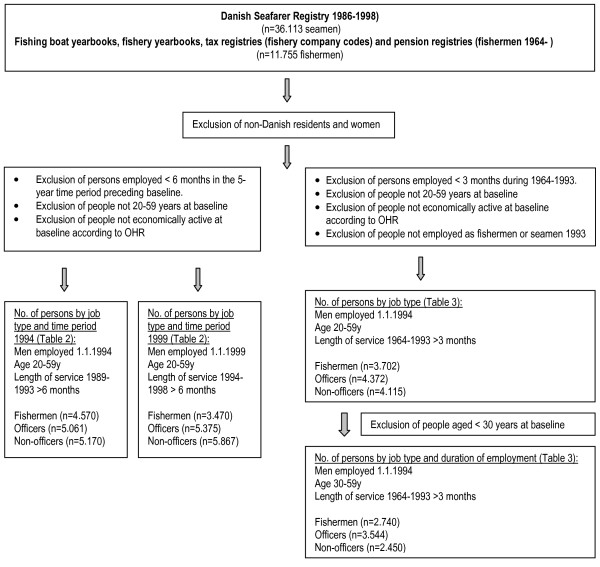
**Selection of the study bases**. Inclusion and exclusion criterias for the occupational cohorts of seafarers and fishermen (identified from registries with detailed information) and linkage to the Occupational Hospitalisation Register (OHR).

Inclusion criteria for follow-up of hospital contacts in the OHR during 1994–1998 were an age between 20 and 59 years on 1 January 1994, Danish residency according to the centralised person register, employment according to the employment classification module, and a total service as a seaman or fisherman of at least six months during 1989–1993 according to the occupational registers. The same inclusion criteria were used for the follow-up of hospital contacts in the OHR in 1999–2003, except that the starting date was 1 January 1999. This cohort consisted of individuals who were employed as seamen or fishermen 1 January 1999 and who had been in these occupations for at least 6 months during 1994–1998. There was a 32% overlap between the two cohorts. A total of 10,231 seamen and 4,570 fishermen were included at baseline 1994 and followed up for diseases in the OHR 1994–1998 and 11,242 seamen and 3,470 fishermen were included at baseline 1999 and followed up for diseases in the OHR 1999–2003 (see Figure 1 and Table 1).

**Table 1:** Number and characteristics of Danish fishermen and seamen at baseline by gender and occupation, men

		Baseline 1 January 1994						Baseline 1 January 1999					
	Job type^1^	Number	Mean age^2 ^Years	Vital status				Number	Mean age^2 ^years	Vital status			
				Alive^3^		Emigrated^4^				Alive^3^		Emigrated^4^	
Characteristics		N		N	%	N	%	N		N	%	N	%
Fishermen	Fishermen	4570	37.2 ± 9.8	4468	97.8	58	1.3	3470	39.5 ± 10.1	3397	97.9	45	1.3
Seamen	Officers	5061	40.2 ± 10.0	4942	97.7	167	3.3	5375	41.3 ± 10.2	5253	97.7	165	3.1
	Non-officers	5170	35.0 ± 11.1	5037	97.4	192	3.7	5867	37.2 ± 11.4	5731	97.7	171	2.9

^1 ^Based on latest held employment before baseline.

^2 ^Mean and standard deviation of age at baseline.

^3 ^Number and percentage of people alive at the end of the follow-up period.

^4 ^Number and percentage of people having emigrated during the follow-up period.

For the follow-up study on hospital contacts in the OHR in the time period 1994–2003 as a function of length of service in the time period 1964–1993 we included those with at least three months of service in the time period 1964–1993. We used the same inclusion condition as above with regard to employment status at the starting date 1 January 1994, and we added the conditions that a person had to be at least 30 years old and employed as a seaman or fisherman some time during 1993 (the year preceding the baseline date) (Figure 1).

### Occupational Hospitalisation Register

The OHR holds information on each individual obtained through record linkage of three Danish national registers: The centralised civil registration system (CRS), the national hospital patient register and the employment classification module. The national hospital patient register has existed since 1977 and contains data from all national public hospitals (more than 99% of all admissions). In the time period 1977–94, the register only included inpatients, but since 1995 it has also included outpatients and emergency ward visits [29]. Since 1994, the diagnoses have been coded according to the International Classification of Diseases, 10^th ^revision (ICD-10). The CRS contains information on gender, addresses and dates of birth, death and migration for every person who is or has been a Danish resident at any time between 1968 and present time. A person's employment status is registered annually in the employment classification module [30]. In Denmark, hospitals services are free of charge and the national health system is tax financed. For most of the population, a hospital is within a driving distance of 30 minutes or less.

### Follow-up for diagnoses in the Occupational Hospitalisation Register

Each person was linked to the files of the nationwide OHR using the PIN. Observation began either on 1 January 1994 or 1 January 1999. The follow-up ended at the date of the hospitalisation for the diagnosis under study, date of death, date of emigration, or at the end of study (31 December 1998 or 31 December 2003), whichever came first. Person-years (PY) at risk were calculated for each individual.

A number of diagnoses were chosen for follow up because previous studies of seamen and fishermen or a recent pilot interview study among Danish fishermen have shown that they may be associated with these occupations [15]. The ICD-10 codes were selected before the study started and included the ICD-10 codes M00–M99 (diseases of the musculoskeletal system and connective tissue), M16 (arthrosis of the hip), M17 (arthrosis of the knee), M51 (thoraco-lumbar disc disorders), M75 (shoulder lesions), M75.1 (rotator cuff syndrome), S00–S99 + T00–T98 (injury, poisoning and certain other consequences of external causes), and G56 (carpal tunnel syndrome). The study was performed in accordance with the requirements of the Ethics Committees in Denmark.

### Statistical analyses

All Danish ships were classified by a code in the registry according to their main use in the periods, e.g. passenger ship, gas tanker or product/chemical tanker. For each 5-year period before start of follow-up, each study participant was assigned to the type of ship on board which he worked during his latest known employment within that 5-year period. Evaluations also took into account the job title of the most recent employment before follow-up which was expected to reflect the seaman's social class membership. Data were only analysed for men.

The SIR was calculated as 100 multiplied by the ratio between the total number of observed cases with a specific hospital diagnosis and the total number of expected cases. The latter was calculated by multiplying the PY at risk during the follow-up period in each five-year age and calendar year group by the corresponding sex-specific rates of hospital contacts among economically active people in the total Danish population. Their corresponding 95% confidence intervals (95% CI) were estimated assuming a Poisson distribution for the observed number of cases with a specific diagnosis. For the SIRs, we calculated exact intervals when the observed number of cases was less than 100. Otherwise, we used the propagation of error formulas and normal approximation to estimate a 95% CI around the logarithm of risk ratio, thereafter transforming it into a 95% CI around the risk ratio. We controlled for age and county at baseline, and for the seamen we also controlled for type of ship (passenger ship versus other).

Length of service was divided into three categories approximately corresponding to tertiles. For fishermen we used the categories 3–71 months, 72–143 months, and >= 144 months based on income data from the pension registry. For seamen we used the categories 3–35 months, 36–71 months, and >= 72 months since the data for seamen was based on actual days at sea and, 6 months at sea correspond to a full income year. The same categories were thus applied for fishermen and seamen. For each occupation, we estimated adjusted relative risks for hospital contacts according to length of service by means of a multiplicative Poisson regression model with no interaction using SAS software version 8.1. We controlled for age and county at baseline and for the seamen we also controlled for type of ship (Passenger ship versus all other categories of ships). We used a likelihood ratio test to test for significance of length of service.

## Results

According to days at sea, the percentage of male seamen with permanent residence in Denmark (as a percentage of all Danish and non-Danish seamen on Danish ships) was 88% for officers, 90% for engineers, 77% for deck crew, 77% for engine crew, 75% for galley and catering crew, and 64% for other professions. Short-term employments were common: 30.6% of the seamen and 41.9% of the fishermen had in 1989–1998 a total length of service of less than 6 months. We therefore made restrictions to more than 6 months of service in the definition of the occupational groups before analyses (figure 1).

Among male fishermen working in 1999, we found an overall high SIR for MSDs, and sub-analysis revealed high SIRs for arthrosis of the knee, thoraco-lumbar disc disorders, shoulder diseases especially rotator cuff syndrome, and injuries. We found high SIR values for carpal tunnel syndrome among fishermen in both the 1994 and the 1999 cohort. The SIR ratio between the two time periods showed a decline in hospital contacts for hip arthrosis among fishermen and an increased risk for injuries in the same group.

Overall, the SIRs for MSD among male seamen working as non-officers did not differ from those seen among economically active men in general. Significantly high SIRs were found in the 1999 cohort for injuries and for carpal tunnel syndrome (Table 2). A sub-analysis revealed that the highest risk for carpal tunnel syndrome was found among non-officers working as deck crew, SIR 233 (95% CI: 166–317) based on 40 cases (data not shown).

**Table 2:** Standardised incidence ratios (SIR) for muskuloskeletal diseases and injuries among male fishermen and seamen in Denmark by job type and time period

	SIR by job type and time period		1994–1998 SIR ^2 ^(4.570 fishermen, 5.061 officers, 5.170 non-officers)			1999–2003 SIR ^3 ^(3.470 fishermen, 5.375 officers, 5.867 non-officers)				
ICD-10^1^	Disease	Job type	Cases	SIR	95% CI	Cases	SIR	95% CI	SIR ratio^4^	95% CI
M00–M99	Diseases of the musculoskeletal system and connective tissue	Fishermen	546	122	113–133	494	118	108–129	0.97	0.86–1.09
		Officers	384	75	68–83	499	75	69–82	1.00	0.87–1.14
		Non-officers	530	107	98–117	730	106	98–114	0.99	0.88–1.11
M16	Arthrosis of the hip – Coxarthrosis	Fishermen	17	162	95–260	5	44	14–103	0.27	0.10–0.73
		Officers	8	51	22–101	12	58	30–101	1.13	0.46–2.76
		Non-officers	5	45	15–106	15	85	48–141	1.89	0.69–5.19
M17	Arthrosis of the knee – Gonarthrosis	Fishermen	30	127	86–181	37	144	101–198	1.13	0.70–1.84
		Officers	28	89	59–129	45	102	74–136	1.14	0.71–1.83
		Non-officers	26	111	73–163	42	110	80–149	0.99	0.61–1.62
M51	Thoraco-lumbar disc disorders	Fishermen	130	224	189–266	78	178	141–222	0.79	0.60–1.05
		Officers	61	91	70–117	63	92	71–118	1.01	0.71–1.43
		Non-officers	65	112	86–143	81	122	97–151	1.09	0.78–1.51
M75	Shoulder lesions	Fishermen	39	162	115–221	50	137	102–181	0.85	0.56–1.29
		Officers	25	87	56–129	43	74	53–99	0.84	0.52–1.38
		Non-officers	24	95	61–141	66	115	89–147	1.22	0.76–1.95
M75.1	Rotator cuff syndrome	Fishermen	20	225	138–348	21	205	127–313	0.91	0.49–1.68
		Officers	9	84	38–159	12	72	37–125	0.86	0.36–2.03
		Non-officers	10	106	51–195	19	119	71–185	1.12	0.52–2.41
S00–S99	Injury, poisoning and certain other consequences of external causes (single body region)	Fishermen	1611	112	107–118	1377	120	114–126	1.07	0.99–1.15
		Officers	1081	68	65–73	1286	67	64–71	0.98	0.90–1.06
		Non-officers	1900	110	105–115	2352	112	108–117	1.02	0.96–1.09
T00–T98	Injury, poisoning and certain other consequences of external causes (more than one body region)	Fishermen	371	98	89–109	357	118	106–130	1.20	1.04–1.39
		Officers	278	72	64–81	316	69	62–77	0.96	0.82–1.13
		Non-officers	565	125	115–136	649	120	111–129	0.96	0.86–1.07
G56	Carpal tunnel syndrome	Fishermen	48	315	233–418	46	292	214–389	0.92	0.62–1.39
		Officers	11	63	31–113	21	85	52–129	1.34	0.65–2.79
		Non-officers	26	168	109–246	42	172	124–233	1.03	0.63–1.67

^1 ^International Classification of Diseases (ICD) version 10.

^2 ^At least 6 months employment 1989–1993. Follow-up of hospital contacts in 1994–1998. SIR adj. for county.

^3 ^At least 6 months employment 1994–1998. Follow-up of hospital contacts in 1999–2003. SIR adj. for county.

^4 ^Calculated if at least 5 cases were expected.

Officers had a statistically significant lower risk of MSD and injuries than economically active men at large.

We observed no statistically significant increase in the occurrence of any of the diseases under study among fishermen for the groups with middle (7 to 12 years) or long duration (more than 12 years) of employment compared with short length of employment (less than 6 years) (Table 3).

**Table 3:** Standardised incidence ratios (SIR) 1994–2003 for muskuloskeletal diseases and injuries among male fishermen and seamen in Denmark by duration of employment

	SIR by job type and duration of employment		Overall SIR adj. county^2 ^(3702 fishermen, 4372 officers, 4115 non-officers)			Short duration^3 ^(1001 fishermen 2252 officers, 1173 non-officers		Med. duration^3,4 ^(1136 fishermen, 1030 officers, 974 non-officers)			Long duration^3,5 ^(603 fishermen, 262 officers, 303 non-officers)			
ICD-10^1^	Site	Job type	Cases	SIR	95% CI	Cases	RR	Cases	RR	95% CI	Cases	RR	95% CI	Prob ChiSq
M00–M99	Diseases of the musculoskeletal system and connective tissue	Fishermen	830	124	116–132	241	1.0	287	1.04	0.87–1.23	122	0.80	0.64–0.99	0.0433
M00–M99		Officers	684	80	74–86	396	1.0	139	0.72	0.59–0.88	49	1.02	0.74–1.39	0.0029
M00–M99		Non-officers	849	111	104–119	292	1.0	209	0.81	0.68–0.97	54	0.61	0.45–0.83	0.0017
M16	Arthrosis of the hip	Fishermen	18	75	44–118	3	1.0	9	2.38	0.65–8.80	6	2.35	0.59–9.39	0.3287
M16		Officers	21	57	35–87	16	1.0	2	-	-	3	-	-	-
M16		Non-officers	14	58	32–98	9	1.0	4	-	-	-	-	-	-
M17	Arthrosis of the knee	Fishermen	69	121	94–153	23	1.0	31	1.09	0.64–1.87	11	0.62	0.30–1.27	0.2311
M17		Officers	63	94	72–120	39	1.0	16	0.84	0.47–1.51	5	0.94	0.35–2.51	0.8381
M17		Non-officers	56	117	88–151	28	1.0	18	0.68	0.37–1.24	3	0.33	0.10–1.12	0.1033
M51	Thoraco-lumbar disc disorders	Fishermen	178	185	160–215	50	1.0	68	1.17	0.81–1.69	21	0.68	0.41–1.13	0.0751
M51		Officers	99	90	74–110	55	1.0	18	0.69	0.40–1.18	12	1.78	0.91–3.48	0.0498
M51		Non-officers	102	110	91–134	43	1.0	22	0.59	0.35–1.00	7	0.57	0.25–1.31	0.0930
M75	Shoulder lesions	Fishermen	80	139	110–172	25	1.0	24	0.81	0.46–1.42	16	0.98	0.52–1.84	0.7240
M75		Officers	57	80	60–103	35	1.0	14	-	-	2	-	-	-
M75		Non-officers	81	137	109–170	34	1.0	23	0.75	0.44–1.29	5	0.51	0.19–1.35	0.2881
M75.1	Rotator cuff syndrome	Fishermen	36	160	112–221	9	1.0	13	-	-	8	-	-	-
M75.1		Officers	19	84	51–131	10	1.0	4	-	-	2	-		-
M75.1		Non-officers	27	143	94–208	11	1.0	9	-	-	2	-	-	
S00–S99	Injury, poisoning and certain other consequences of external causes (single body region)	Fishermen	1999	140	134–146	517	1.0	609	1.04	0.93–1.17	259	0.83	0.72–0.96	0.0066
S00–S99		Officers	1574	71	68–75	766	1.0	368	1.05	0.93–1.19	79	0.89	0.70–1.13	0.3799
S00–S99		Non-officers	2206	109	105–114	583	1.0	445	0.92	0.82–1.05	124	0.77	0.63–0.95	0.0375
T00–T98	Injury, poisoning and certain other consequences of external causes (more than one body region)	Fishermen	569	143	132–155	155	1.0	151	0.87	0.69–1.08	67	0.77	0.58–1.02	0.1629
T00–T98		Officers	445	75	68–82	220	1.0	83	0.84	0.65–1.09	27	1.17	0.77–1.78	0.2454
T00–T98		Non-officers	753	122	113–131	216	1.0	135	0.76	0.61–0.95	32	0.57	0.39–0.84	0.0028
G56	Carpal tunnel syndrome	Fishermen	88	267	214–328	29	1.0	27	0.78	0.46–1.32	12	0.73	0.37–1.44	0.5497
G56		Officers	28	70	47–102	19	1.0	8	-	-	1	-	-	-
G56		Non-officers	56	180	136–234	24	1.0	13	-	-	6	-	-	-

^1 ^International Classification of Diseases (ICD) version 10.

^2 ^In the calculation of the overall SIR we included people who were >= 20 years.

^3^In the analysis of risk as a function of duration of employment we included people who were >= 30 years. Short duration of employment corresponding to less than 6 years of income was used as a reference.

^4 ^Medium duration of employment corresponding to 6–12 years of income.

^5 ^Long duration of employment corresponding to more than 12 years of income.

## Discussion

Among male fishermen in 1999, we found an increased occurrence of injuries and MSD, and sub analysis showed increased SIRs for arthrosis of the knee, shoulder diseases, and carpal tunnel syndrome. Non-officer seamen had high SIRs for injuries and carpal tunnel syndrome, whereas officers had low SIRs. Long term cumulative effects of employment were not shown for any of the disease outcomes possibly suggesting a healthy worker effect.

The main strength of the present study is that it is based on a comparison between an old and a more recent cohort of seamen with the same type of employment details. Equally valid information was obtained for fishermen owing to the successful creation of a reliable fishermen cohort through a combination of several registries.

The possible disadvantages of the study were a high turn-over of the cohort members and the lack of data on specific exposures. Approximately two thirds of the hospital contacts were outpatient visits. The level did not differ between seafarers and the population at large. The lack of information about outpatients in 1994 may have decreased the number of cases with MSD in this year, but this would be the case for both the seafarers and the rest of the Danish population (our comparison group). We have decided to keep the information on out-patients in the statistics, because we believe it gives valuable information. The number of Danish fishermen declined with 25% between 1994 and 1999 as seen in Table 1. Since we only included fishermen and seamen with an active employment in 1994 and 1999, respectively, we do not expect that this have changed the results.

We used the economically active Danish population as a reference group in order to reduce a healthy worker effect. Referral bias may constitute a problem in studies using hospital admissions as a measure of disease, but especially for diseases commonly treated outside hospitals, e.g. back pain, and for diseases for which people are only admitted to hospital if it progresses to a severe disease [31]. We do not expect to have eliminated a healthy worker selection bias by using other employed people as a reference. Selection into the workforce is job-specific and the healthy worker effect may have been further enhanced by the obligatory health examinations for seamen. We therefore expect our results to be biased towards low estimates for some of the diseases we studied. Admission of seamen and fishermen to hospitals in other countries may take place for acute diseases and accidents and low risks are therefore not easily interpreted. We expect that Danish seamen and fishermen are generally referred to treatment at Danish hospitals since medical treatment is free in Denmark. Hospitalisation abroad that is not followed by hospitalisation in Denmark will bias risk estimate towards null. The threshold for hospital contacts generally differs within a population, and we expect people working at sea to have a higher threshold for hospitalisation for most of the diseases studied in this paper due to their long working periods. Still, we found an increased incidence of hospitalisation due to injuries among both fishermen and non-officers. Among fishermen, the SIRs for injuries were increased in 1999 and the SIR ratio for hospital contacts for these conditions rose between the two time periods.

Despite pre-employment selection, we found high hospitalisation rates in the 1999 cohort for knee arthrosis and lumbar disc disorders among fishermen compared with economically active men in general, but that may be due to a difference in the hospitalisation threshold over time. Biomechanical analysis of fishermen on fishing vessels from Sweden revealed that ship motions were mainly counteracted by motions in the lower extremity and lumbar back and induced increased strain here when subjects were standing erect [32]. Holding a load considerably increased the pressure on most joints. Other papers have shown that symptoms from the musculoskeletal system were common and correlated with age, number of years in the fishing trade, type of fishing and type of work on board [20,21,33]. Work tasks implying severe workload included handling the fishing gear and handling the catch. In a Swedish study, musculoskeletal symptoms were one of the major reasons for leaving the job as a fisherman [34]. All fishermen in Denmark have access to compensation if they are forced to quit their job due to work-related illness, especially back pain after many years of heavy lifting. Our finding of arthrosis of the knee among fishermen, but not among seamen, may be due to differences in the size of the ships, the relative stability of the ship at sea, work tasks or employment time. Injuries or a high body-mass index may also be a risk factor for arthrosis of the knee among fishermen, but our data did not allow us to examine this hypothesis [9]. Since 2002–2003 it has in Denmark also been compulsory for fishermen to pass the health examinations, and the requirements of "fit for duty" are now the same as for seamen. Back in time it is possible that the situation was different. An earlier study of 299 seamen referred to an orthopaedic clinic for knee pathology found that 51% of these patients had a diagnosis of knee osteoarthritis [35]. Genu varus or bow legs were present in 31% of the seamen studied.

Our finding of a high SIR for shoulder diseases among fishermen is backed by data from a recent study showing that shoulder crepitation tended to be more common among fishermen than among welders [10,14]. The findings of a nearly 2-fold elevated SIRs for carpal tunnel syndrome and rotator cuff syndrome among fishermen are new. These associations were present in both cohorts and may be real. A high incidence of traumatic injuries to the shoulder or wrist during fishery or in the spare time may partly explain the finding, but our data did not allow us to investigate this hypothesis further, since only major injuries is expected to be treated at hospitals. Fishermen are also known to have repetitive hyperflexing and twisting movements of the wrists in cold surroundings (when doing repair on the net or tearing fish out of the net), which may explain the wrist symptoms, and physically demanding repetitive work with their shoulder elevated during work with ice in the hold may explain the damage to the shoulder tendons.

Male non-officers had increased SIRs for injuries as shown in previous studies. An increased incidence of injuries compared with the general population indicates that injuries and lesions remain a problem in this trade, even for modern ships [36]. Officers, on the other hand, have less physically demanding work, and we would therefore expect rates for these diseases to be lower and at levels corresponding to those encountered for white collar workers on shore. The increased SIR for carpal tunnel syndrome among non-officers working as deck crew may be due to work with hand-vibrating tools on board (during corrosion repair work) [17]. Other possible explanations were former injuries of the wrist, other working processes or exposures in the spare time.

## Conclusion

Fishermen still have increased SIRs for injuries and MSDs and the SIR was augmented for injuries and reduced for hip arthrosis between the two time periods. Our findings suggest an association between work within fishery and the incidence of shoulder lesions, rotator cuff syndrome and carpal tunnel syndrome. Among non-officers the SIRs for injuries and carpal tunnel syndrome were high. A sub-analysis revealed that the highest risk for carpal tunnel syndrome was found among male non-officers working as deck crew. Officers have low SIRs for injuries and MSDs. The number of employed Danish fishermen declined with 25% between 1994 and 1999 to 3470. Short-term employments are common in the trades. None of the SIRs increased with increasing length of employment in any of the three trades. Whether these diseases were caused by physically demanding repetitive work, injuries or other conditions among fishermen and seamen needs to be confirmed in studies with more detailed exposure data and confounding control.

## Competing interests

The author(s) declare that they have no competing interests.

## Authors' contributions

All authors (LK, AJ, PSN, JO, HH, FT) participated in the design and coordination of the study. LK applied for funding, carried out the data collection, prepared the dataset for statistical analyses and drafted the manuscript. JO helped to draft the manuscript. HH, FT and LK performed the statistical analysis. All authors (LK, AJ, PSN, JO, HH, FT) read, commented and approved the manuscript.

The national research Centre for the Working Environment contributed reference data from the Occupational Hospitalisation register collected and organized by Elsa Bach, FT, and HH

## Pre-publication history

The pre-publication history for this paper can be accessed here:



## References

[B1] Jaremin B, Kotulak E, Starnawska M, Mrozinski W, Wojciechowski E (1997). Death at sea: certain factors responsible for occupational hazard in Polish seamen and deep-sea fishermen. Int J Occup Med Environ Health.

[B2] Jensen OC, Stage S, Noer P, Kaerlev L (2003). Risk assessment in fishery. Classification of working processes to facilitate occupational hazard coding on industrial trawlers. Am J Ind Med.

[B3] Jensen OC (1996). Mortality in Danish fishermen. Bull Inst Marit Trop Med Gdynia.

[B4] Jensen OC (1996). Work related injuries in Danish fishermen. Occup Med.

[B5] Wagner B (2003). Safety and health in the fishing industry. Int Marit Health.

[B6] Thomas TK, Lincoln JM, Husberg BJ, Conway GA (2001). Is it safe on deck? Fatal and non-fatal workplace injuries among Alaskan commercial fishermen. Am J Ind Med.

[B7] Grainger CR (1993). Hazards of commercial fishing. World Health Forum.

[B8] Fugelli P, Toft JJ (1984). [Do fishermen often suffer from illness? Health problems in fishermen]. Tidsskr Nor Laegeforen.

[B9] Matheson C, Morrison S, Murphy E, Lawrie T, Ritchie L, Bond C (2001). The health of fishermen in the catching sector of the fishing industry: a gap analysis. Occup Med (Lond).

[B10] Torner M, Zetterberg C, Anden U, Hansson T, Lindell V (1991). Workload and musculoskeletal problems: a comparison between welders and office clerks (with reference also to fishermen). Ergonomics.

[B11] Filikowski J, Rzepiak M, Renke W (1998). Health problems of deep sea fishermen. Bull Inst Marit Trop Med Gdynia.

[B12] Filikowski J, Krynicki A (1979). Estimation of the health condition of deep-sea fishermen based on examination of their actual morbidity. Bull Inst Marit Trop Med Gdynia.

[B13] Casson FF, Zucchero A, Boscolo Bariga A, Malusa E, Veronese C, Boscolo Rizzo P, Chiereghin F, Boscolo Panzin C, Mancarella P, Mastrangelo G (1998). Work and chronic health effects among fishermen in Chioggia, Italy. G Ital Med Lav Ergon.

[B14] Lipscomb HJ, Loomis D, McDonald MA, Kucera K, Marshall S, Li L (2004). Musculoskeletal symptoms among commercial fishers in North Carolina. Appl Ergon.

[B15] Kærlev L Fiskeri og helbred Et pilot studie.

[B16] Svendsen SW, Bonde JP, Mathiassen SE, Stengaard-Pedersen K, Frich LH (2004). Work related shoulder disorders: quantitative exposure-response relations with reference to arm posture. Occup Environ Med.

[B17] Palmer KT, Harris EC, Coggon D (2007). Carpal tunnel syndrome and its relation to occupation: a systematic literature review. Occup Med (Lond).

[B18] Videman T, Levälahti E, Battié MC (2007). The effects of anthropometrics, lifting strength, and physical activities in disc degeneration. Spine.

[B19] Lau EC, Cooper C, Lam D, Chan VN, Tsang KK, Sham A (2000). Factors associated with osteoarthritis of the hip and knee in Hong Kong Chinese: obesity, joint injury, and occupational activities. Am J Epidemiol.

[B20] Torner M, Blide G, Eriksson H, Kadefors R, Karlsson R, Petersen I (1988). Workload and ergonomics measures in Swedish professional fishing. Appl Ergon.

[B21] Torner M, Blide G, Eriksson H, Kadefors R, Karlsson R, Petersen I (1988). Musculo-skeletal symptoms as related to working conditions among Swedish professional fishermen. Appl Ergon.

[B22] Jensen O, Sorensen JFL, Kaerlev L, Canals ML, Nikolic N, Saarni H (2004). Self-reported injuries among seamen. Questionnaire validity and results from an international study. Accid Anal Prev.

[B23] Kaerlev L, Hansen J, Hansen HL, Nielsen PS (2005). Cancer incidence among Danish seamen – A population-based cohort study. Occup Environ Med.

[B24] Hansen HL, Tüchsen F, Hannerz H (2005). Hospitalization among seamen on merchant ships. Occup Environ Med.

[B25] Kaerlev L, Dahl S, Nielsen PS, Olsen J, Hannerz H, Jensen A, Tüchsen F (2007). Hospital contacts for chronic diseases among Danish seamen and fishermen – A population-based cohort study. Scand J Pub Health.

[B26] Kaerlev L, Jensen A, Nielsen PS, Olsen J, Hannerz H, Tüchsen F (2008). Hospital contacts for noise related hearing loss among Danish seafarers and fishermen – A population-based cohort study. Noise and Health.

[B27] Hansen HL (1996). Occupation-related morbidity and mortality among merchant seamen with particular reference to infectious diseases.

[B28] Silver Star Reklame, Denmark (2000). Danmarks Kutternøgle Skagen.

[B29] Tüchsen F, Bach E, Marmot M (1992). Occupation and hospitalization with ischaemic heart diseases: a new nationwide surveillance system based on hospital admissions. Int J Epidemiol.

[B30] Soll-Johanning H, Hannerz H, Tuchsen F (2004). Referral bias in hospital register studies of geographical and industrial differences in health. Dan Med Bull.

[B31] Tuchsen F, Andersen O, Olsen J (1996). Referral bias among health workers in studies using hospitalization as a proxy measure of the underlying incidence rate. J Clin Epidemiol.

[B32] Torner M, Almstrom C, Karlsson R, Kadefors R (1994). Working on a moving surface – a biomechanical analysis of musculo-skeletal load due to ship motions in combination with work. Ergonomics.

[B33] Torner M, Zetterberg C, Hansson T, Lindell V, Kadefors R (1990). Musculo-skeletal symptoms and signs and isometric strength among fishermen. Ergonomics.

[B34] Torner MI, Nilsson E, Kadefors R (1990). The influence of musculo-skeletal load, and other factors, on staff turn-over in fishery: a post employment questionnaire study. Bull Inst Marit Trop Med Gdynia.

[B35] Pearce MS, Buttery YE, Brueton RN (1996). Knee pathology among seamen: a review of 299 patients. Occup Med (Lond).

[B36] Hansen HJ, Nielsen D, Frydenberg M (2002). Occupational accidents aboard merchant ships. Occup Environ Med.

